# Supplementary material for: Harnessing liquid-in-liquid printing and micropatterned substrates to fabricate 3-dimensional all-liquid fluidic devices
Source: Nat Commun. 2019 Mar 6;10:1095. doi: 10.1038/s41467-019-09042-y (PMC6403306; doi:10.1038/s41467-019-09042-y)
Supplement: Supplementary file 1 — Supplementary Information [file 41467_2019_9042_MOESM1_ESM.pdf]

Supplementary Information

for

Harnessing Liquid-in-Liquid Printing and Micropatterned Substrates to Fabricate 3-Dimensional All-Liquid Fluidic Devices

Feng *et al.*

Supplementary Figures

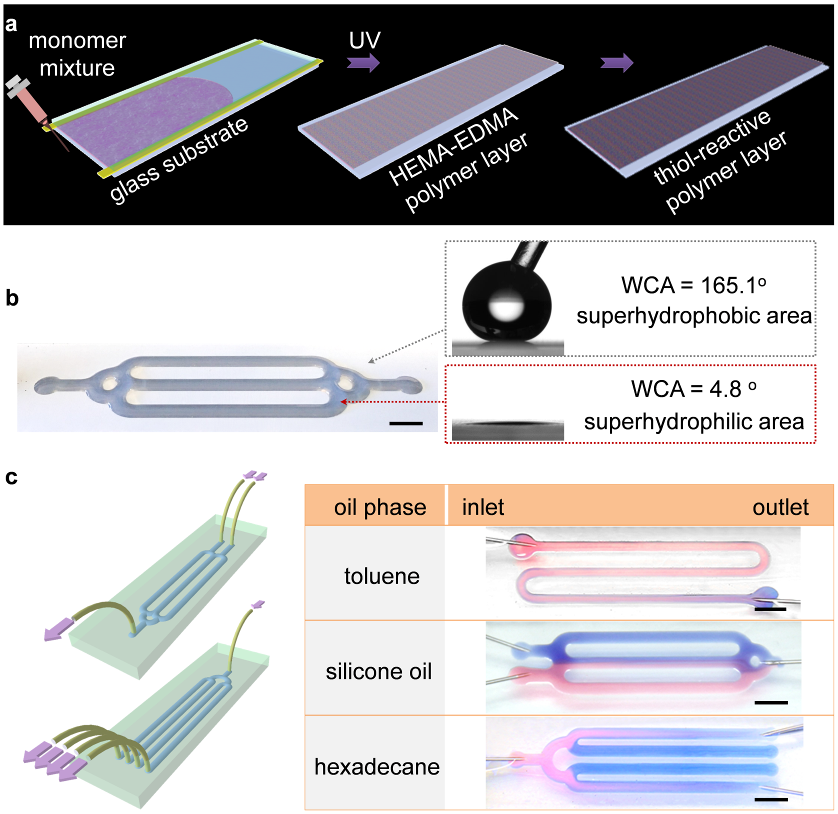

Supplementary Figure 1 | Diversification of all-liquid fluidic architectures using superhydrophobic-superhydrophilic micropatterned substrates and various water–oil pairings. a, A 25 μm-thin polymer layer on a glass substrate was prepared using photoinitiated co-polymerization of 2-hydroxyethyl methacrylate (HEMA) and ethylene dimethacrylate (EDMA). The polymer layer was then esterified with 4-pentynoic acid to give a surface functionalized with alkynes. Superhydrophobic-superhydrophilic micropatterns were subsequently created via UV-induced sequential thiol–yne click chemistry on this thiol-reactive polymer layer. **b,** Photograph of a superhydrophobic-superhydrophilic pattern filled with nanoclay dispersion (10 mg mL^{-1}). Water droplets on a superhydrophobic surface (*upper*) and on a superhydrophilic surface (*lower*) show the corresponding water contact angles (WCA). In air, the nanoclay dispersion only wets the superhydrophilic areas to fill in the pattern's contour, while the superhydrophobic areas remain dry. **c**, Aqueous solutions containing dye are injected into the inlets and withdrawn from the outlets by external pumps through hollow needles at 1 mL h^{-1}. Scale bars, 5 mm.

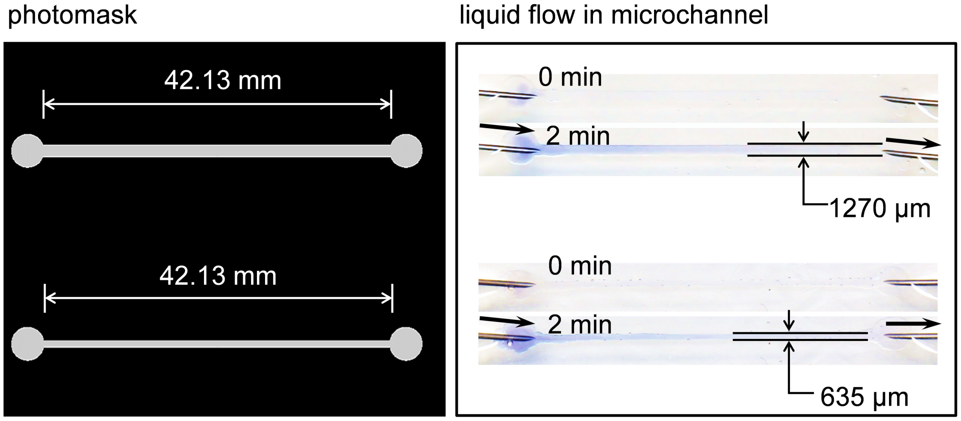

Supplementary Figure 2 | Demonstrating flow through fluidic channels of varying width. Time-lapse showing an aqueous solution of resazurin dye (50 μg mL^{-1}, pH 7.0) pumped through the liquid channels with widths of 1270 μm and 635 μm at a flow rate of 0.5 mL h^{-1}.

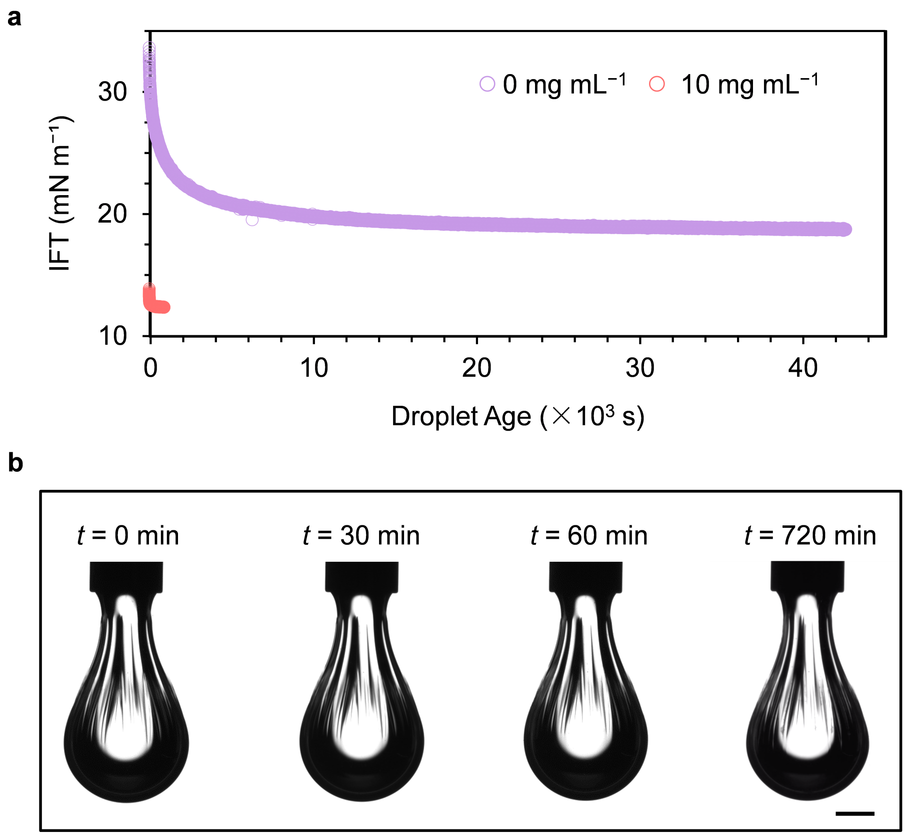

Supplementary Figure 3 | Self-forming nanoclay–polymer surfactant assembly at the liquid–liquid interface. a, Temporal evolution of the interfacial tension (IFT) of an aqueous droplet, either loaded with the 2-D nanoclay (10 mg mL^{-1}, pH 7.0) (**red**) or not (**purple**), suspended in a solution of NH_2-PDMS-NH_2 in toluene (10% *w*/*w*), differentiating the rates at which the system reaches steady-state and the extent to which nanoclay–polymer surfactants further decrease IFT when compared to the polymer surfactant layer on its own. Whereas an aqueous droplet suspended in toluene containing the polymer reached an equilibrium IFT of 18.9 mN m^{-1} over 10 h; the nanoclay–polymer system reached steady-state with IFT of 12.8 mN m^{-1} within 60 s. **b,** Snapshots over 12 h of the nanoclay–polymer interfacial film's irreversible buckling behavior, observed after retracting a cladded droplet that had reached steady-state with respect to IFT. Scale bar: 1 mm.

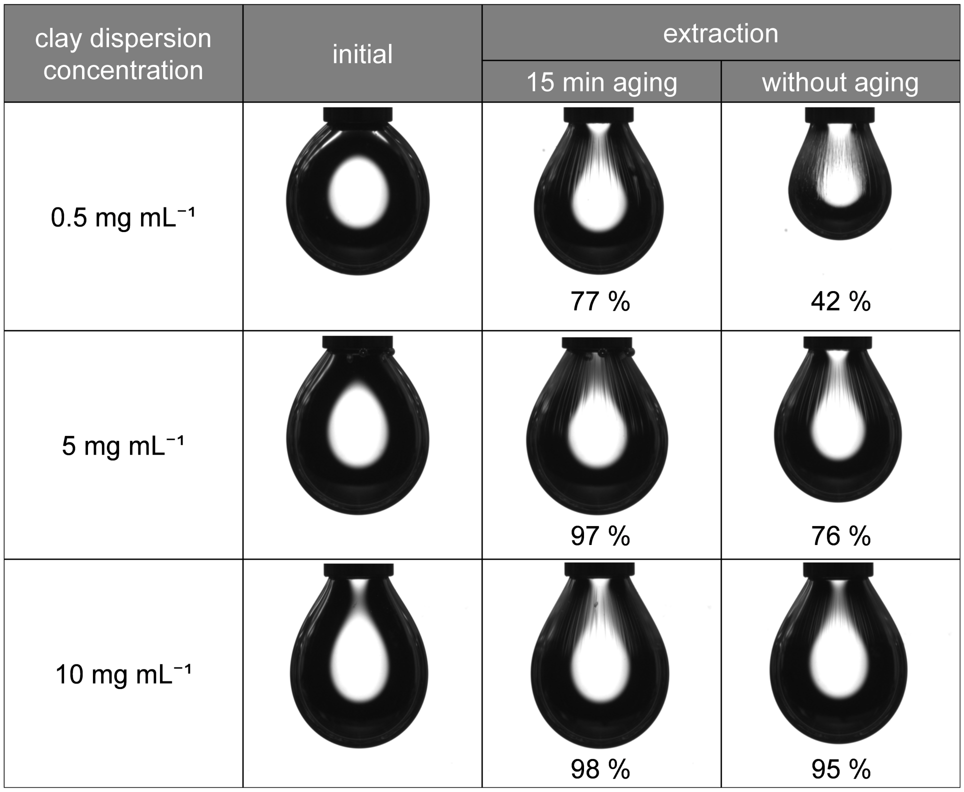

Supplementary Figure 4 | Droplet morphologies of nanoclay dispersions (0.5, 5 and 10 mg mL^{-1}, pH 7.0) introduced to solutions of NH_2–PDMS–NH_2 in toluene (10 % *w/w*) and NPS buckling behavior when the nanoclay dispersion was withdrawn after 15 min of aging or immediately withdrawn without aging. The onset of wrinkling upon droplet retraction is indicated as a percentage of the initial droplet volume. The onset of visible wrinkling defines the point at which the nanoclay assembly at the liquid–liquid interface is densely packed, and this volume ratio defines the amount that the assembly of interface area must be compressed to solidify the assembly.

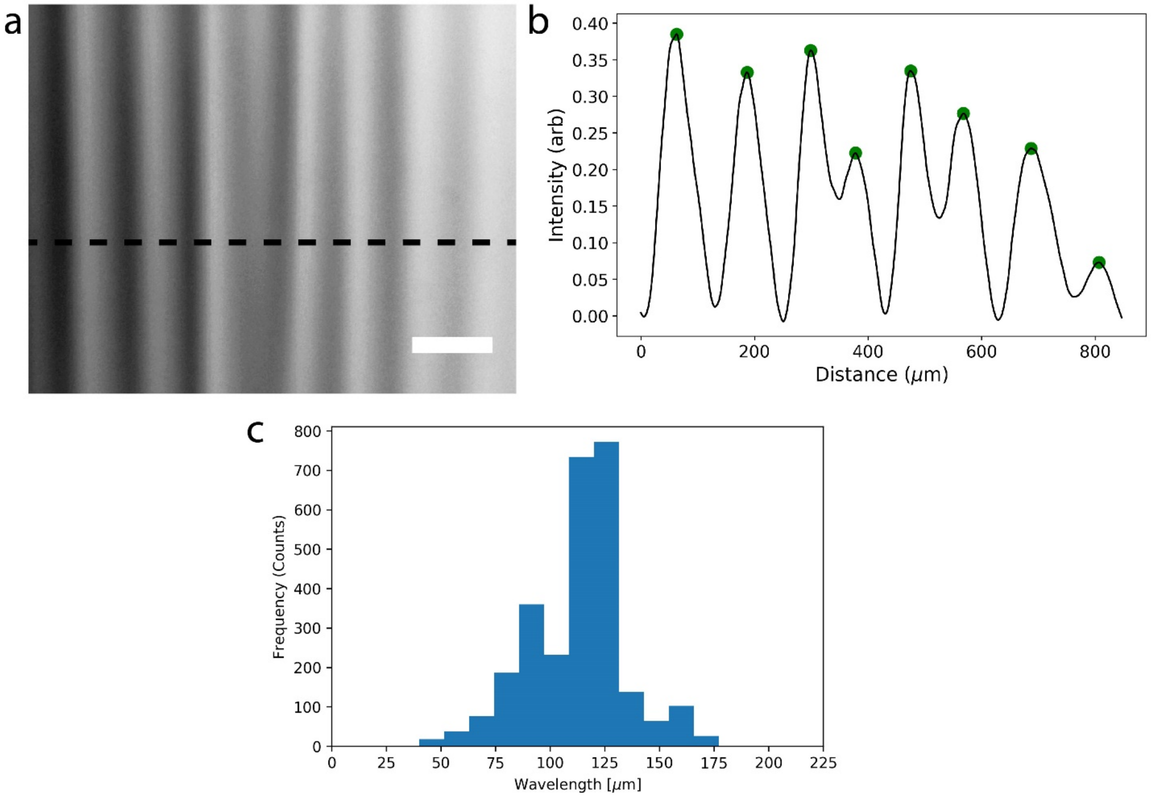

Supplementary Figure 5 | Measuring the wavelength of NPS wrinkles. a, Micrograph of wrinkles in a NPS film at the water–oil interface. **b**, Intensity profile measured along the dashed line in (**a)** after subtraction of a polynomial background**.** Green markers indicate the location of detected peaks**. c**, Histogram showing measured wrinkle wavelengths.

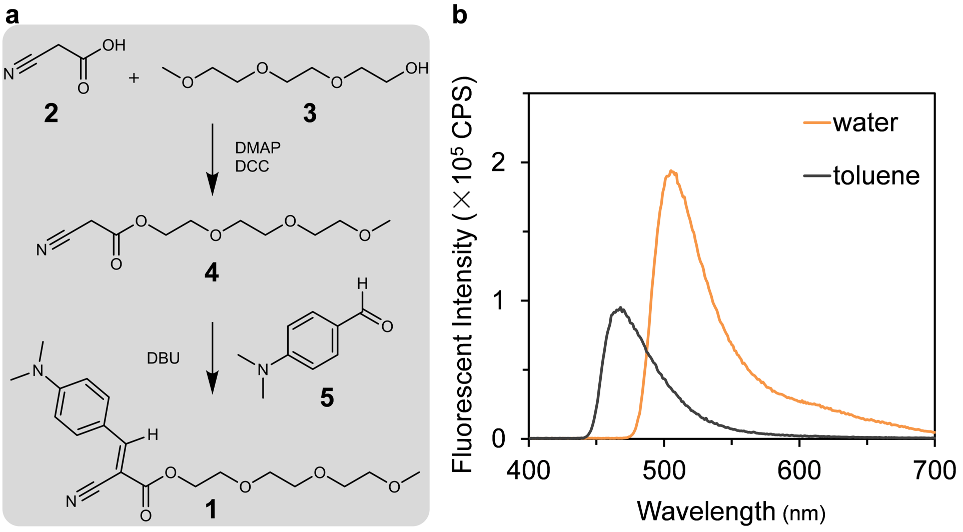

Supplementary Figure 6 | Synthesis and optical properties of ambipolar dye 1. a, Schematic of the synthesis of fluorescent dye, (*E*)-2-(2-(2-methoxyethoxy)ethoxy)ethyl 2-cyano-3-(4-(dimethylamino)phenyl)acrylate (**1**). **b**, Fluorescence emission spectra of **1** in water (0.2 mg mL^{-1}) and in toluene (0.2 mg mL^{-1}). Excitation wavelength: λ = 380 nm.

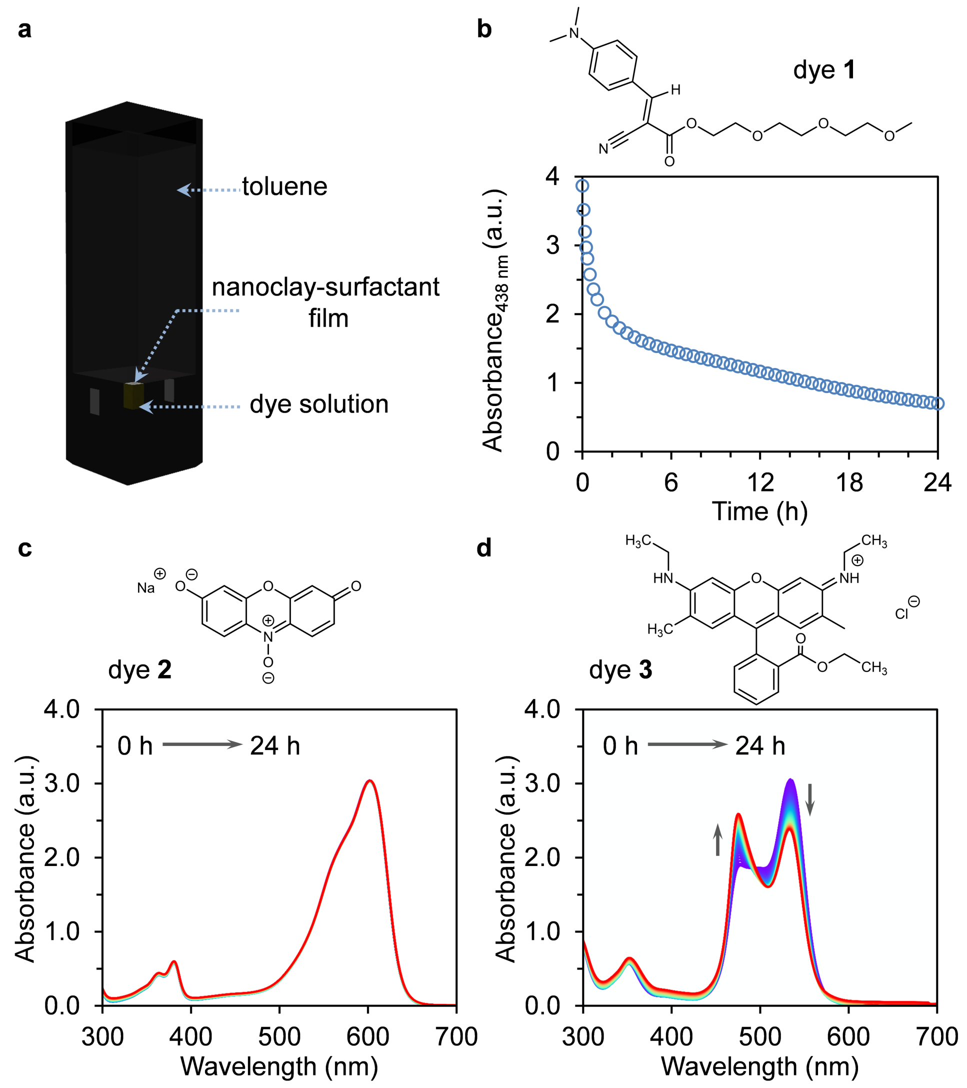

Supplementary Figure 7 | Permeability of NPS films. **a**, Schematic diagram of the experimental setup for monitoring dye transfer from water to toluene through the NPS film in a masked cuvette. **b,** Evolution of the absorbance of aqueous phase containing dye **1** (non-ionic molecule) at λ = 438 nm. **c,** Stacked UV–vis spectra of the aqueous phase containing resazurin (anionic dye **2**) in the masked cuvette for 24 h. **d,** Stacked UV–vis spectra of the aqueous phase containing rhodamine 6G (cationic dye **3**) in the masked cuvette for 24 h.

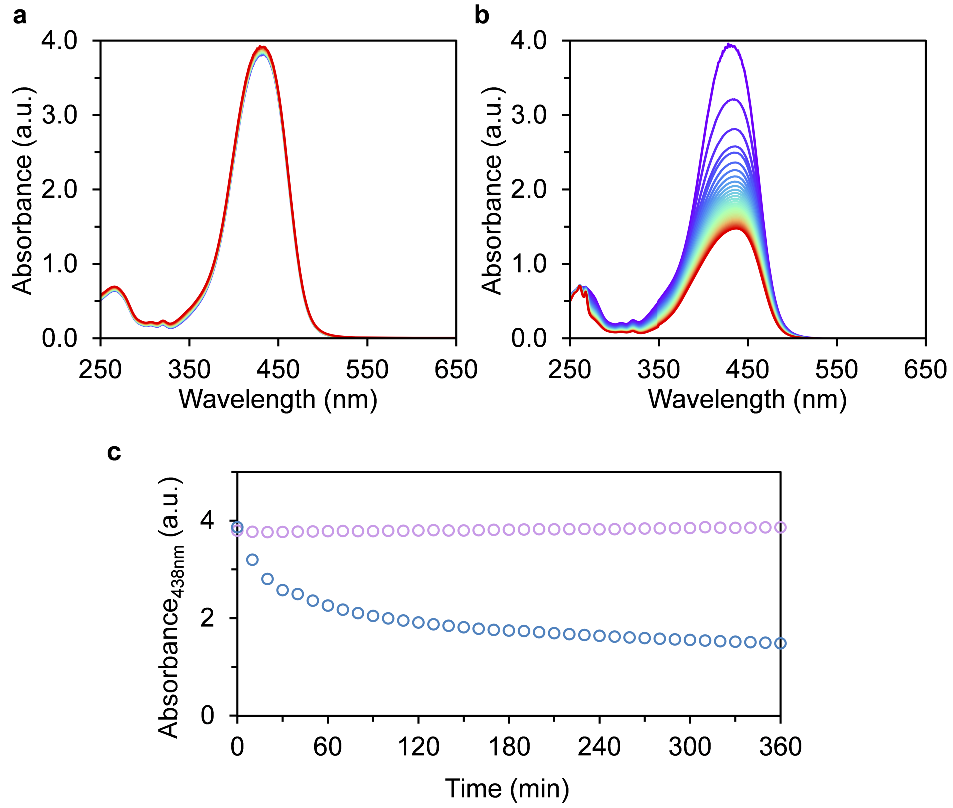

Supplementary Figure 8 | Tracking non-ionic dye 1 in the aqueous phase. a, Stacked UV–vis spectra of non-ionic dye **1** at 10 min time-intervals in the absence of the oil phase. Neither the accumulation of dye **1** on the dispersed nanoclays nor dye quenching was observed over 6 h. **b,** Stacked UV–vis spectra of non-ionic dye **1** at 10 min time-intervals with oil phase, monitoring dye transfer from water to toluene across the NPS membrane film in the masked cuvette. **c,** Evolution in absorbance at λ = 438 nm for an aqueous phase containing non-ionic dye **1** in systems configured with (**blue**) or without (**purple**) the oil phase, illustrating dye partitioning from water to toluene across the self-assembled nanoclay–polymer interfacial film.

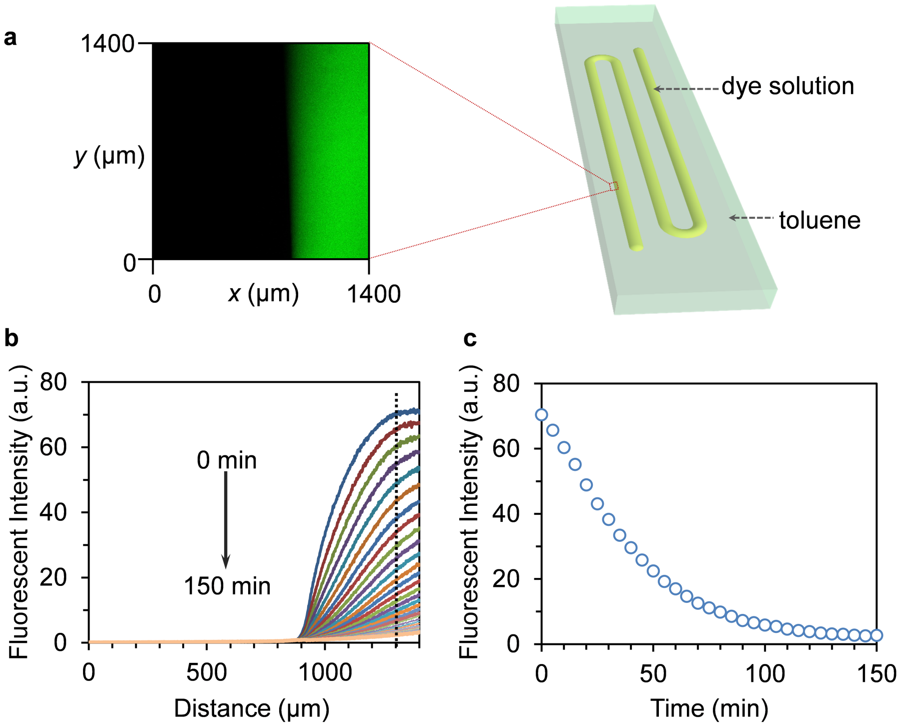

Supplementary Figure 9 | Direct monitoring of mass transfer from water to toluene through NPS film in an all-liquid fluidic system. a, Top-Down 2D fluorescence image of the fluidic channel's edge, obtained by LCSM. This is defined as the initial state of the system (0 min). **b**, Stacked intensity profiles of the fluorescence along the *x*-axis at 5 min time intervals. **c**, Evolution of the fluorescent intensity at x = 1300 μm with time.

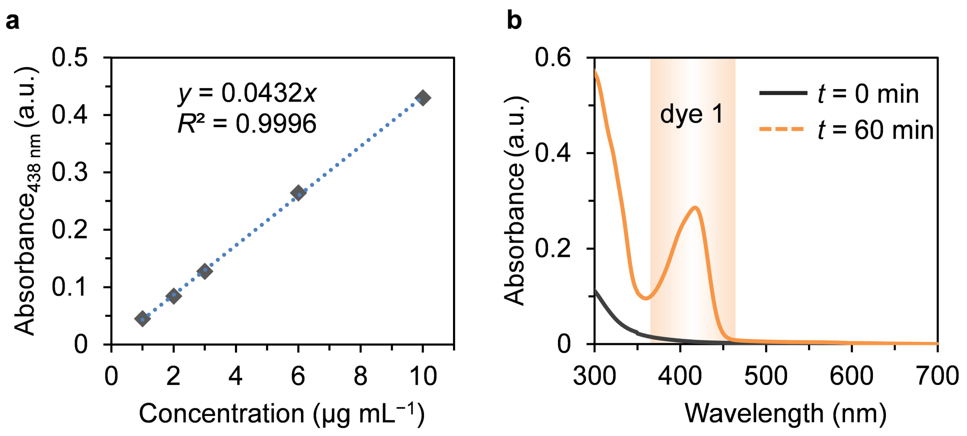

Supplementary Figure 10 | Dye isolation in an all-liquid fluidic channel. a, Standard calibration of ambipolar dye **1** in water obtained by UV–vis spectrophotometer at 438 nm. **b**, UV–vis spectra of the overlays of toluene solutions before and after infusing dye solution for 60 min.

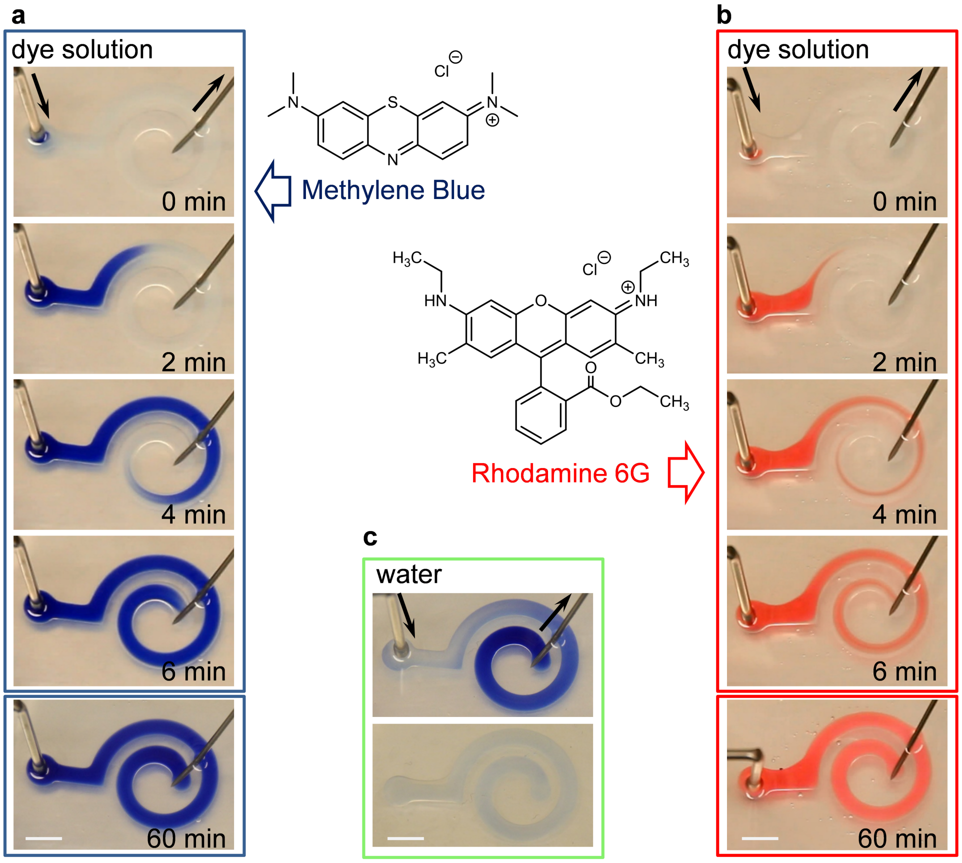

Supplementary Figure 11 | Anionic NPS films at the liquid–liquid interface serve as supports for immobilizing cationic small molecules. a and **b**, Time-lapse showing solutions of methylene blue (**a**) and rhodamine 6G (**b**) flowing through NPS-stabilized liquid channels at a flow rate of 0.5 mL h^{-1}. **c**, Purging a channel with water for 30 min at a flow rate of 0.5 mL h^{-1}. Scale bars, 5 mm.

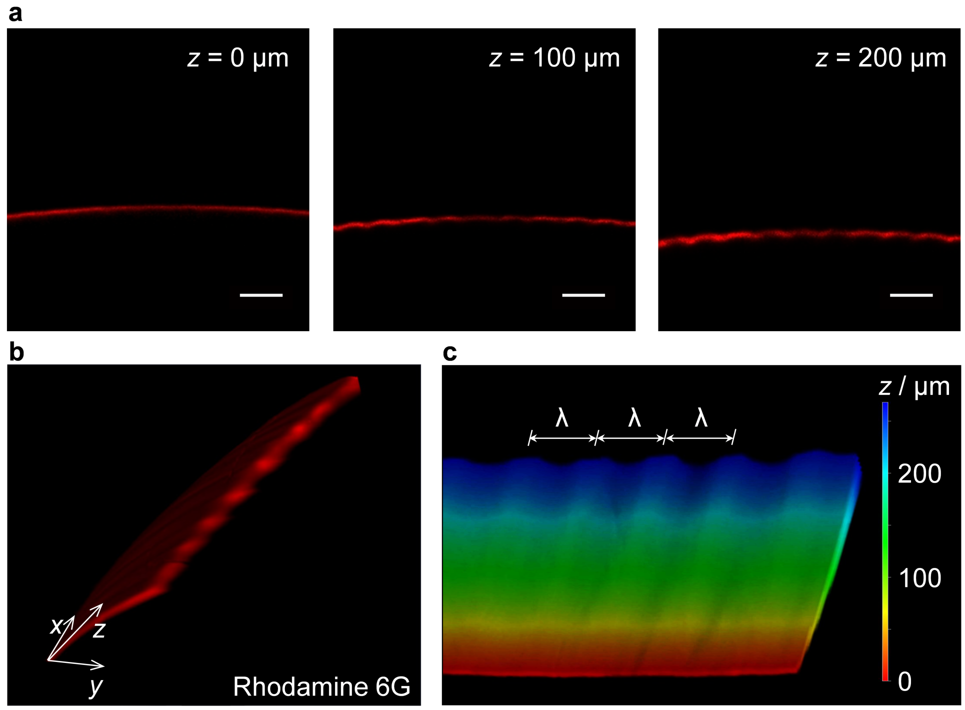

Supplementary Figure 12 | Visualizing the immobilization of cationic fluorescent rhodamine 6G to the anionic NPS wall in the all-liquid device. a, LSCM images of the channel after infusing the dye solution and washing as illustrated in Supplementary Fig. 11. The images show the optical cross-sectioning of the NPS wall at different depths. The fluorescence from rhodamine 6G is shown in red (excitation, 514 nm; detection range, 521–699 nm). Scale bar, 100 μm. **b**, 3D fluorescent image of rhodamine 6G@NPS constructed through a series of *z*–stack confocal microscopy images. The curved, shell-shaped distribution of rhodamine 6G suggests the successful anchoring of dye to the NPS wall after they have been assembled. **c**, Wrinkles of the NPS assembly as a result of volume loss of the aqueous phase. This 3D fluorescent image of the rhodamine 6G is displayed according to the *z* depths. Wavelength of the wrinkles (λ) is 150 ± 4 μm calculated from measuring 8 wrinkles.

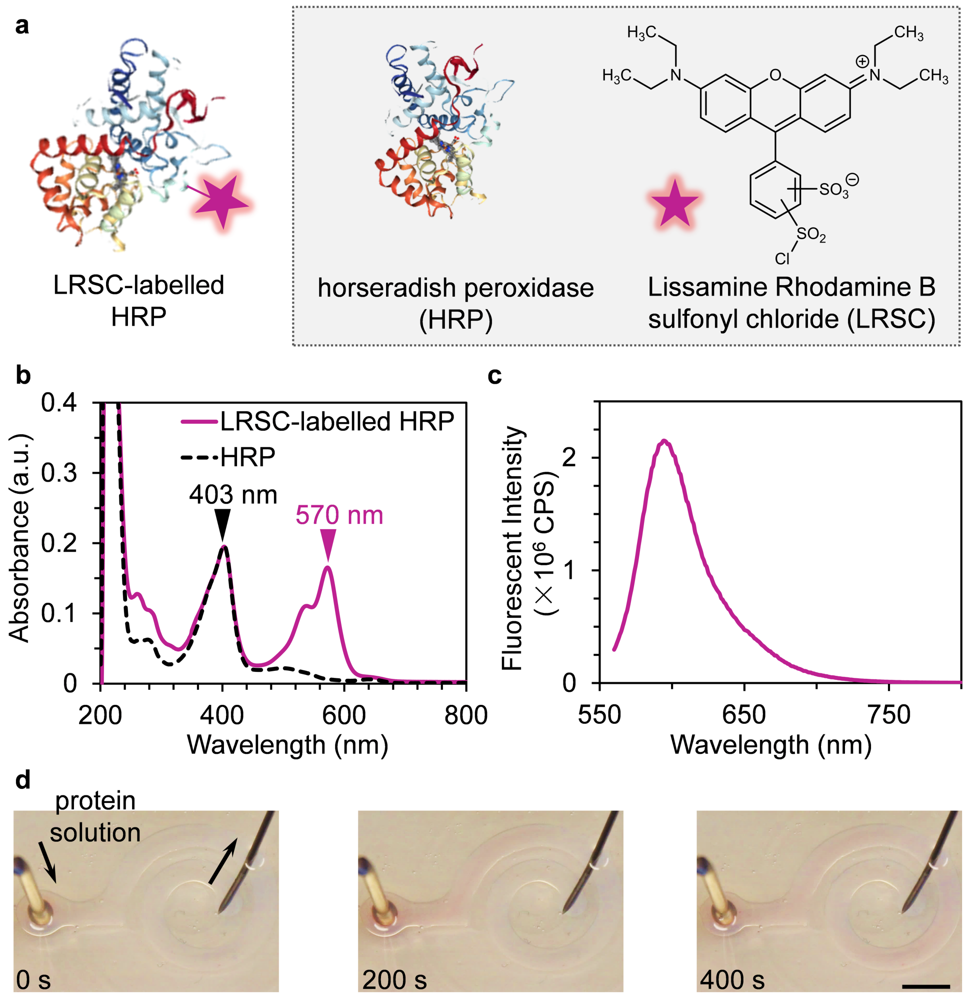

Supplementary Figure 13 | Anionic NPS films at the liquid–liquid interface serve as a support on which cationic enzymes may be immobilized. a, LRSC-labeled HRP. **b**, Normalized absorption of HRP before (black, dashed line) and after (pink, solid line) LRSC labelling. **c**, Fluorescence emission spectra of the LRSC-labelled HRP (~1 mg mL^{-1}) in MES buffer. **d**, Infusing 100 μg mL^{-1} of LRSC-labeled HRP in MES buffer (pH 6.5) through the liquid channel at a flow rate of 0.5 mL h^{-1}. Scale bar, 5 mm. Imaging the channel with LSCM was performed after washing the channel with pure buffer. Scale bar, 5 mm.

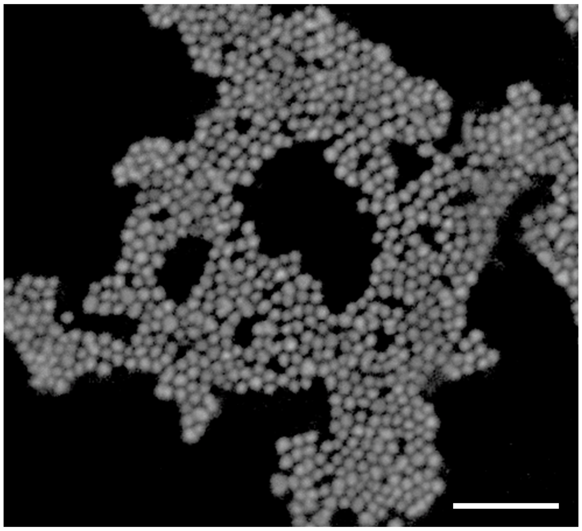

Supplementary Figure 14 | SEM image of CTAB-coated Pt nanocrystals. Scale bar, 100 nm.

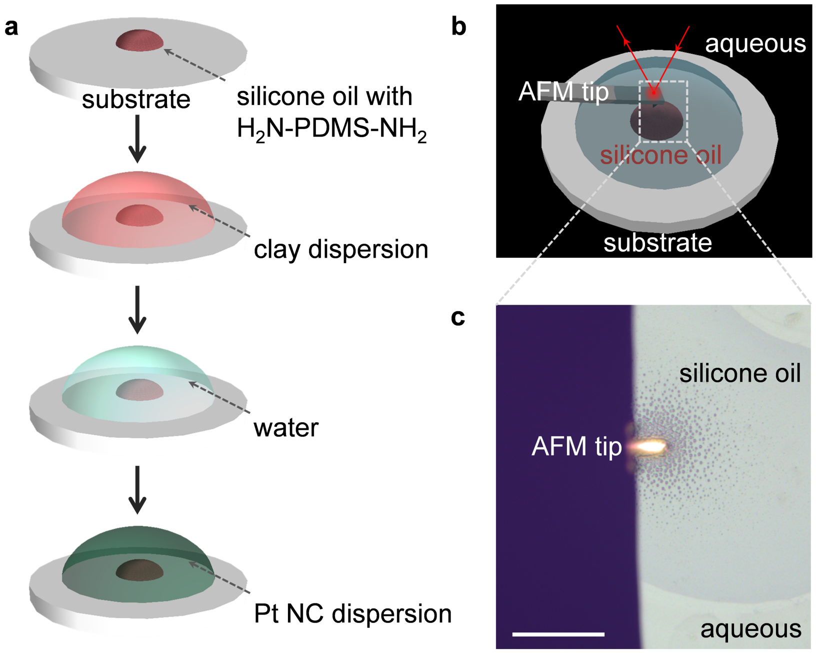

Supplementary Figure 15 | Anionic NPS films at the liquid–liquid interface serve as supports for immobilizing CTAB-coated colloidal nanocrystals to the walls of the all-liquid fluidic devices. **a**, Immobilizing CTAB-coated Pt NCs onto the NPS assembly at liquid–liquid interface for i*n-situ* AFM imaging. **b**, Schematic diagram of the experimental setup for *in-situ* AFM. **c**, A photograph of the AFM tip being brought into contact with the water–oil interface while imaging. Scale bar, 100 µm.

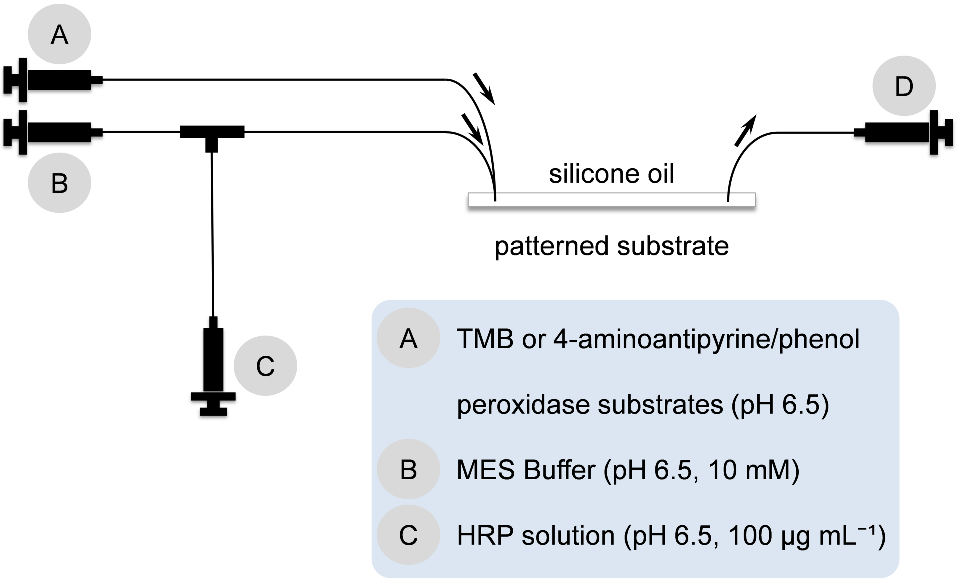

Supplementary Figure 16 | Experimental setup for utilizing the all-liquid fluidic device as a continuous-flow enzymatic microreactor. To investigate the activity of immobilized catalysts for carrying out chemical transformations involving complex organic molecules, two chromogenic reactions catalyzed by peroxidases were devised and tested; oxidation of 3,3',5,5'-tetramethylbenzidine (TMB), and oxidative coupling of 4-AAP with phenol; both use H_2O_2 as the stoichiometric oxidant. Both HRP and CTAB-coated Pt NCs have peroxidase activity.[1] To understand which had the highest activity, we conducted a comparative study of the oxidative coupling of 4-aminoantipyrine (4-AAP) and phenol (5 mM 4-AAP, 25 mM phenol, 50 mM H_2O_2) (Supplementary Fig. 17). The superior activity of HRP was put to work in our proof-of-concept all-liquid microreactor-on-a-chip demonstrations.

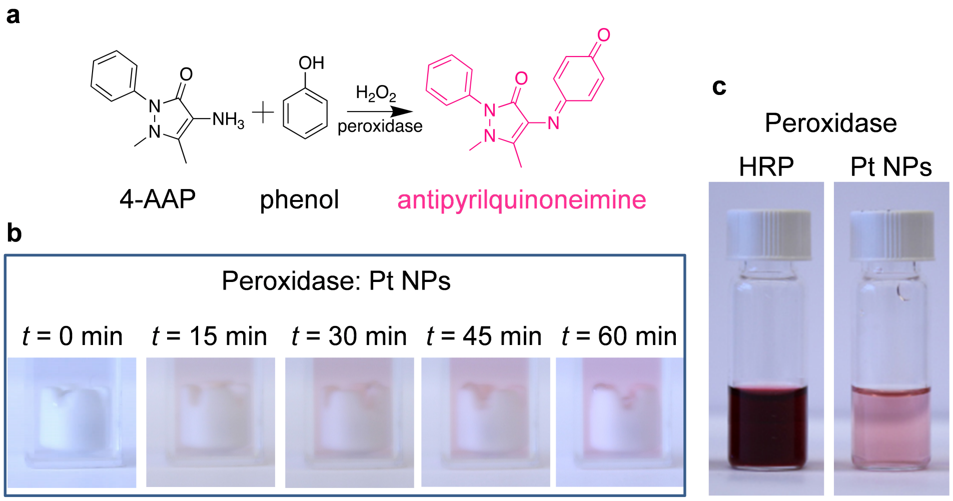

Supplementary Figure 17 | Peroxidase-like activity of CTAB-coated Pt nanoparticles. a, Schematic of the oxidative coupling of 4-AAP and phenol, catalyzed by natural and artificial peroxidases. **b,** Color change of a 4-AAP/phenol/H_2O_2 solution (5 mM 4-AAP, 25 mM phenol, 50 mM H_2O_2) over time after adding 40 µL of Pt NPs suspension (~100 µg mL^{-1}). **c,** Photos of a 4-AAP/phenol/H_2O_2 solution (5 mM 4-AAP, 25 mM phenol, 50 mM H_2O_2) after adding 4 µL of HRP (100 µg mL^{-1}, pH 6.5, left photo) over 1 min or after adding 40 µL of Pt NPs suspension (~100 µg mL^{-1}, right photo) over 60 min, showing the superior activity of HRP relative to CTAB-coated Pt nanoparticles in this reaction. Pt nanocrystal catalysis in solution in no way correlates to the Pt catalysis in an all-liquid fluidic device, and thus the activity may be different.

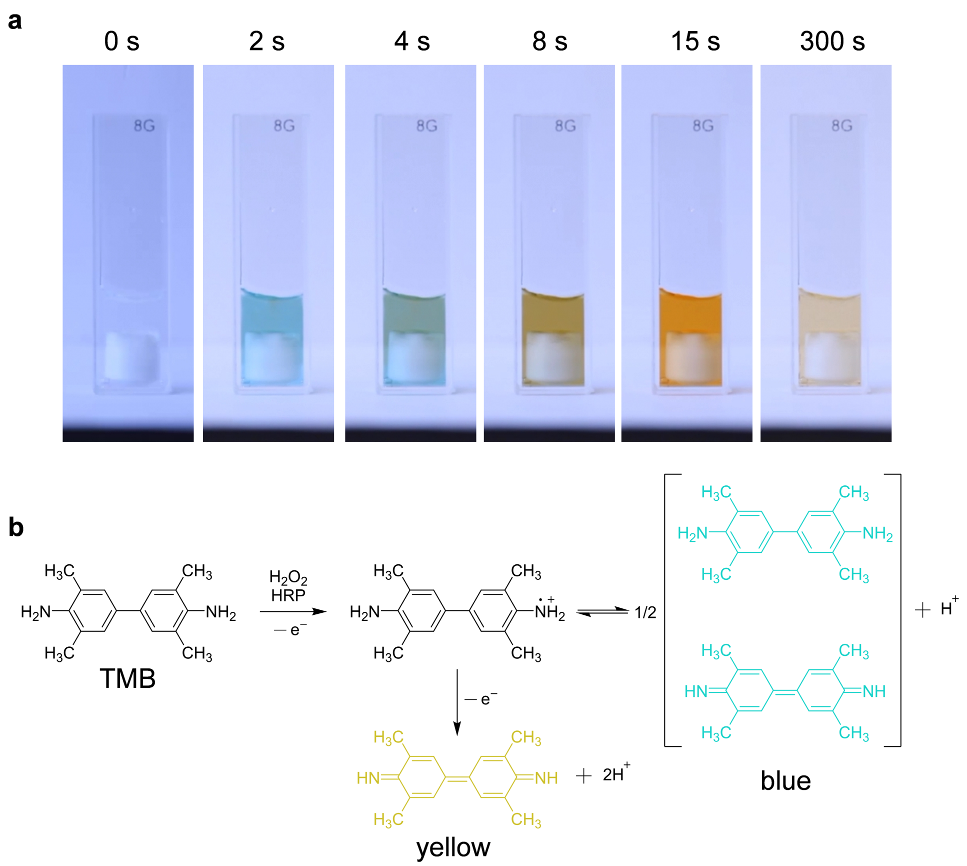

Supplementary Figure 18 | HRP-catalyzed conversion of TMB to various colored products. **a,** Color change of the TMB solution over time after adding 4 μL of HRP in MES buffer (100 μg mL^{-1}, pH 6.5). Incubations turn blue, pass through a green stage, and finally become yellow. A gradual fading of the yellow diimine product can occur with time, especially in neutral buffers.[2] **b**, Schematic representation of two-step TMB conversion in the presence of HRP and H_2O_2.

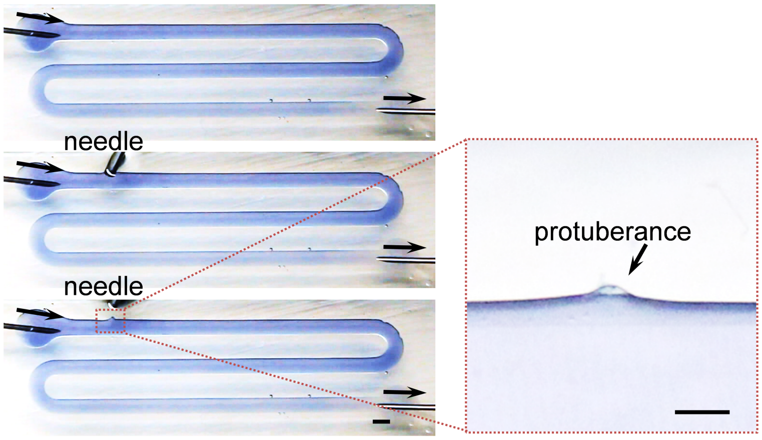

Supplementary Figure 19 | Self-healing properties of NPS interfacial films. When the microchannel under flow (3 mL h^{-1}) is punctured by a needle, the protuberance at the damaged part is visibly shape-persistent after the needle removal, illustrating the speed in which new NPSs form at the liquid–liquid interface. Scale bars, 2 mm in left panel and 500 μm in right panel.

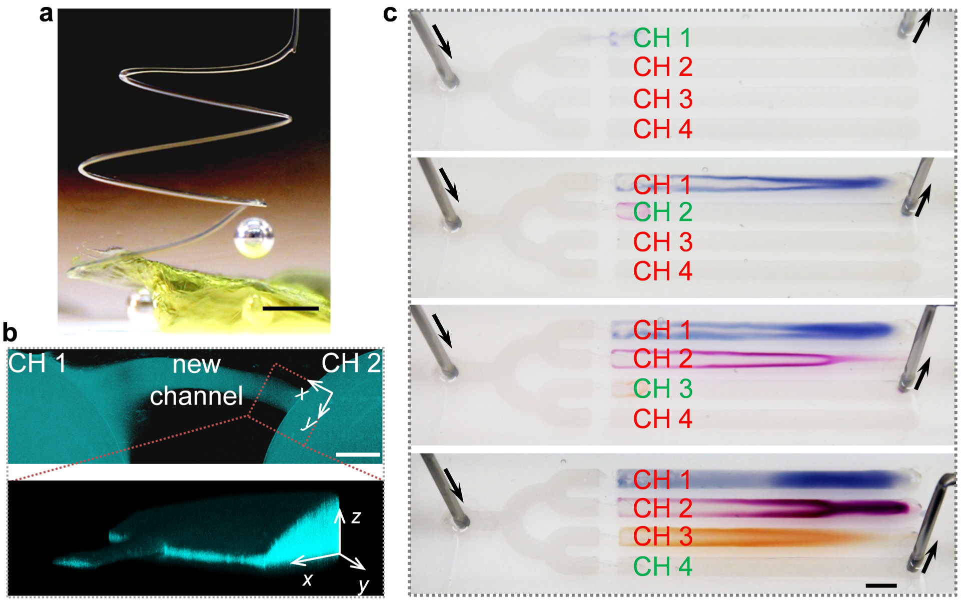

Supplementary Figure 20 | Spatial and temporal control of various chromogenic reactions in the fluid channels under flow, toward a new type of chemical logic where the history of the device's flow paths is indicated by specific colors and patterns of these colors in space over time. a, 3D printed spiral threads allow patterned regions to be coupled to pumps and sensors without being spatially confined to the underlying substrate. Scale bar: 2 mm. **b**, 3D reconstitution of LSCM images of printed liquid bridge between two patterned fluid channels loaded with an aqueous solution of methylene blue (CH 2). Scale bar: 500 µm. **c**, Consecutive oxidative coupling reactions in three channels of an all-liquid fluidic device, yielding chromogenically distinct products in the different patterned regions defined by channels 1–3. All-liquid "bridges" were printed to connect the inlet stream to the different channels containing various reactants. After completing each reaction in the various fluid channels, the bridge was clipped to prevent chemical contamination. Scale bar: 5 mm.

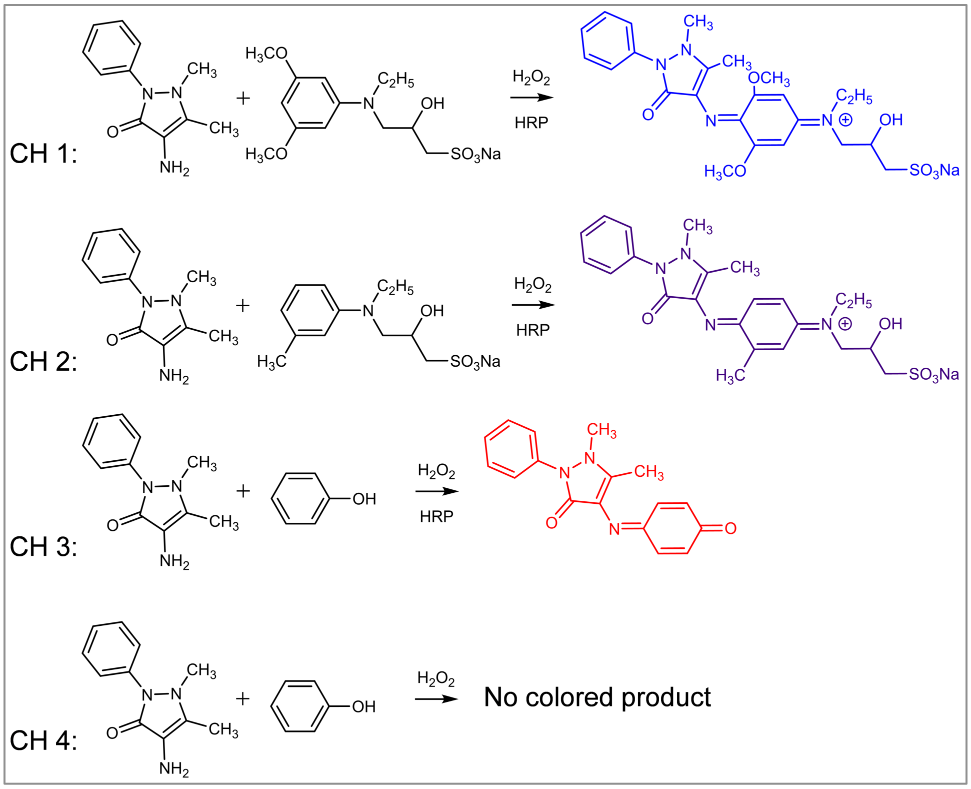

Supplementary Figure 21 | Schematic representation of the molecular transformations in each channel.

Supplementary References

1. Ma, M., Zhang, Y. & Gu, N. Peroxidase-like catalytic activity of cubic Pt nanocrystals. *Colloids Surf. A: Physicochemical and Engineering Aspects* **373**, 6–10, (2011).

2. Rye, D. B., Saper, C. B. & Wainer B. H. Stabilization of the tetramethylbenzidine (TMB) reaction product: application for retrograde and anterograde tracing, and combination with immunohistochemistry. *J. Histochem. Cytochem.* **32,** 1145–1153 (1984).
