# Supplementary material for: Harnessing liquid-in-liquid printing and micropatterned substrates to fabricate 3-dimensional all-liquid fluidic devices
Source: Nat Commun. 2019 Mar 6;10:1095. doi: 10.1038/s41467-019-09042-y (PMC6403306; doi:10.1038/s41467-019-09042-y)
Supplement: Supplementary file 3 — Description of Additional Supplementary Files [file 41467_2019_9042_MOESM3_ESM.pdf]

Description of Additional Supplementary Files

Supplementary Movie 1

Description: Wrinkling of the pendant drop during injection of a nanoclay dispersion into and subsequent withdrawal from toluene containing surfactant at a rate of 0.2 mL min^{-1}, showcasing rapid NPS interfacial assembly.

Supplementary Movie 2

Description: Pumping aqueous dye at a flow rate of 10 mL h^{-1} through micropatterned liquid-in-liquid channels, with or without the NPS wall present.

Supplementary Movie 3

Description: Mass transfer across the NPS wall while pumping aqueous dye through the micropatterned liquid-in-liquid channel.

Supplementary Movie 4

Description: Continuous-flow liquid-in-liquid microreactor-on-a-chip for in-line catalyzed chemical transformations.
